# mHealth Intervention for Promoting Hypertension Self-management Among African American Patients Receiving Care at a Community Health Center: Formative Evaluation of the FAITH! Hypertension App

**DOI:** 10.2196/45061

**Published:** 2023-06-16

**Authors:** LaPrincess C Brewer, Clarence Jones, Joshua P Slusser, Maarya Pasha, Mathias Lalika, Megan Chacon, Patricia Takawira, Stanton Shanedling, Paul Erickson, Cynthia Woods, Ashton Krogman, Daphne Ferdinand, Paul Underwood, Lisa A Cooper, Christi A Patten, Sharonne N Hayes

**Affiliations:** 1 Department of Cardiovascular Medicine Mayo Clinic College of Medicine Rochester, MN United States; 2 Center for Health Equity and Community Engagement Research Mayo Clinic Rochester, MN United States; 3 Hue-Man Partnership Minneapolis, MN United States; 4 Department of Quantitative Health Sciences Division of Clinical Trials and Biostatistics Mayo Clinic Rochester, MN United States; 5 Department of Internal Medicine Hennepin Healthcare Minneapolis, MN United States; 6 Cardiovascular Health Unit Minnesota Department of Health St. Paul, MN United States; 7 NorthPoint Health and Wellness Center Minneapolis, MN United States; 8 Open Cities Health Center St. Paul, MN United States; 9 Healthy Heart Community Prevention Project, Inc. New Orleans, LA United States; 10 Interventional Cardiology/Structural Heart Boston Scientific Corporation Marlborough, MA United States; 11 Department of Medicine Johns Hopkins University School of Medicine Baltimore, MD United States; 12 Department of Psychiatry and Psychology Mayo Clinic College of Medicine Rochester, MN United States

**Keywords:** African Americans, hypertension, telemedicine, health promotion, community health workers, community-based participatory research, mobile phone, mobile health

## Abstract

**Background:**

African American individuals are at a higher risk of premature death from cardiovascular diseases than White American individuals, with disproportionate attributable risk from uncontrolled hypertension. Given their high use among African American individuals, mobile technologies, including smartphones, show promise in increasing reliable health information access. Culturally tailored mobile health (mHealth) interventions may promote hypertension self-management among this population.

**Objective:**

This formative study aimed to assess the feasibility of integrating an innovative mHealth intervention into clinical and community settings to improve blood pressure (BP) control among African American patients.

**Methods:**

A mixed methods study of African American patients with uncontrolled hypertension was conducted over 2 consecutive phases. In phase 1, patients and clinicians from 2 federally qualified health centers (FQHCs) in the Minneapolis-St Paul, Minnesota area, provided input through focus groups to refine an existing culturally tailored mHealth app (Fostering African-American Improvement in Total Health! [FAITH!] App) for promoting hypertension self-management among African American patients with uncontrolled hypertension (renamed as FAITH! Hypertension App). Phase 2 was a single-arm pre-post intervention pilot study assessing feasibility and patient satisfaction. Patients receiving care at an FQHC participated in a 10-week intervention using the FAITH! Hypertension App synchronized with a wireless BP monitor and community health worker (CHW) support to address social determinants of health–related social needs. The multimedia app consisted of a 10-module educational series focused on hypertension and cardiovascular risk factors with interactive self-assessments, medication and BP self-monitoring, and social networking. Primary outcomes were feasibility (app engagement and satisfaction) and preliminary efficacy (change in BP) at an immediate postintervention assessment.

**Results:**

In phase 1, thirteen African American patients (n=9, 69% aged ≥50 years and n=10, 77% women) and 16 clinicians (n=11, 69% aged ≥50 years; n=14, 88% women; and n=10, 63% African American) participated in focus groups. Their feedback informed app modifications, including the addition of BP and medication tracking, BP self-care task reminders, and culturally sensitive contexts. In phase 2, sixteen African American patients were enrolled (mean age 52.6, SD 12.3 years; 12/16, 75% women). Overall, 38% (6/16) completed ≥50% of the 10 education modules, and 44% (7/16) completed the postintervention assessment. These patients rated the intervention a 9 (out of 10) on its helpfulness in hypertension self-management. Qualitative data revealed that they viewed the app as user-friendly, engaging, and informative, and CHWs were perceived as providing accountability and support. The mean systolic and diastolic BPs of the 7 patients decreased by 6.5 mm Hg (*P*=.15) and 2.8 mm Hg (*P*=.78), respectively, at the immediate postintervention assessment.

**Conclusions:**

A culturally tailored mHealth app reinforced by CHW support may improve hypertension self-management among underresourced African American individuals receiving care at FQHCs. A future randomized efficacy trial of this intervention is warranted.

**Trial Registration:**

ClinicalTrials.gov NCT04554147; https://clinicaltrials.gov/ct2/show/NCT04554147

## Introduction

### Background

Cardiovascular disease (CVD) accounts for the deaths of nearly 1 in 5 adults in the United States annually [1,2]. There are striking CVD disparities, as African American individuals are 30% more likely to die from CVD than non-Hispanic White individuals [3,4]. The prevalence of hypertension, a major modifiable CVD risk factor, in the African American population is among the highest in the world [5-9]. Recent US data show that 56.6% of African American men and 55.3% of African American women have hypertension and are less likely than non-Hispanic White individuals to achieve blood pressure (BP) control [2]. Uncontrolled hypertension is associated with heightened cardiovascular morbidity and mortality and the use of health care resources. African American individuals in Minnesota are no exception, with their CVD and hypertension patterns mirroring national statistics, along with greater premature CVD mortality rates than those of White Minnesotans [10,11].

These marked health disparities can in part be attributed to adverse social determinants of health (SDOH), which are more prevalent among African American individuals, such as increased stress due to racism or discrimination [12], poor education or employment opportunities, scant access to nutritional foods, and impoverished living conditions [13-18]. Underresourced populations face further impediments, such as insufficient access to transportation, limiting their reach to preventive care. They may also lack awareness of the link between hypertension and CVD and how hypertension can be effectively self-managed [19-21]. Together, these factors can lead to poor health outcomes. Poor adherence to self-care behaviors (medication, regular physical activity [PA], low-salt diet, and weight loss or maintenance) may also contribute to hypertension disparities among African American individuals [22]. African American individuals have lower adherence to BP medications and are less likely to follow a low-sodium, Dietary Approaches to Stop Hypertension (DASH) diet than non-Hispanic White individuals with hypertension [2,23]. African American women are the least physically active racial or ethnic group in the United States; thus, they are at a high risk for obesity (a hypertension risk factor) [24]. Evidence suggests that increasing their self-efficacy or confidence in their ability to manage their BP may predict adherence to self-care behaviors among African American individuals with hypertension. Thus, encouraging self-care activities can assist in reducing hypertension disparities [22,25].

In 2020, the US Department of Health and Human Services issued a call to action to make hypertension control a national priority through the implementation of evidence-based interventions in diverse settings in the United States [26]. This will require effective, multipronged, and patient-centered interventions with rapid dissemination potential to simultaneously address negative SDOH and uncontrolled hypertension among African American patients. Patients in federally qualified health centers (FQHCs) are particularly susceptible to poor health outcomes due to the high prevalence of uncontrolled hypertension [27]. Furthermore, there are large racial disparities in hypertension control between African American and non-Hispanic White individuals at FQHCs [28]. A potential opportunity to mitigate these poor hypertension outcomes comes from the observation that African American individuals are increasingly using mobile technologies, such as smartphones, as their sole means of accessing health information, which may provide vehicles to deliver culturally tailored mobile health (mHealth) tools [29,30]. These technologies are cost-effective and have the potential for widespread dissemination to bolster patient engagement with health care teams and self-management of chronic disease [31]. There is also evidence suggesting that digital health tools could be used by community health workers (CHWs) and can assist in providing health education, resources, and referrals to address unmet social needs and determinants of health [32]. CHWs help bridge cultural and linguistic barriers and act as a crucial link between the community and health care system by providing outreach, health education, care coordination, and advocacy for the patients they serve [33-36]. CHW-integrated interventions have been shown to improve BP control in FQHCs and other low-resource settings among African American patients [19,37]. These findings provide convincing evidence that culturally tailored interventions using mHealth technologies with adjunct CHW support have the potential to improve BP control in a meaningful way.

### An Innovative Intervention to Improve Hypertension Self-management

Building upon our longstanding community-based participatory research (CBPR) partnership [38], we conducted a mixed methods study to refine and test an existing community-, cardiovascular health (CVH) promotion–, mHealth-, and app-based intervention to improve hypertension self-management among African American patients. The intervention incorporates a patient-CHW-clinician triad to work collaboratively in addressing hypertension disparities and SDOH-related social needs at local FQHCs. We hypothesized that our intervention would demonstrate feasibility and preliminary efficacy in improving BP among African American patients with uncontrolled hypertension.

## Methods

### Study Design Overview

**Figure 1:**
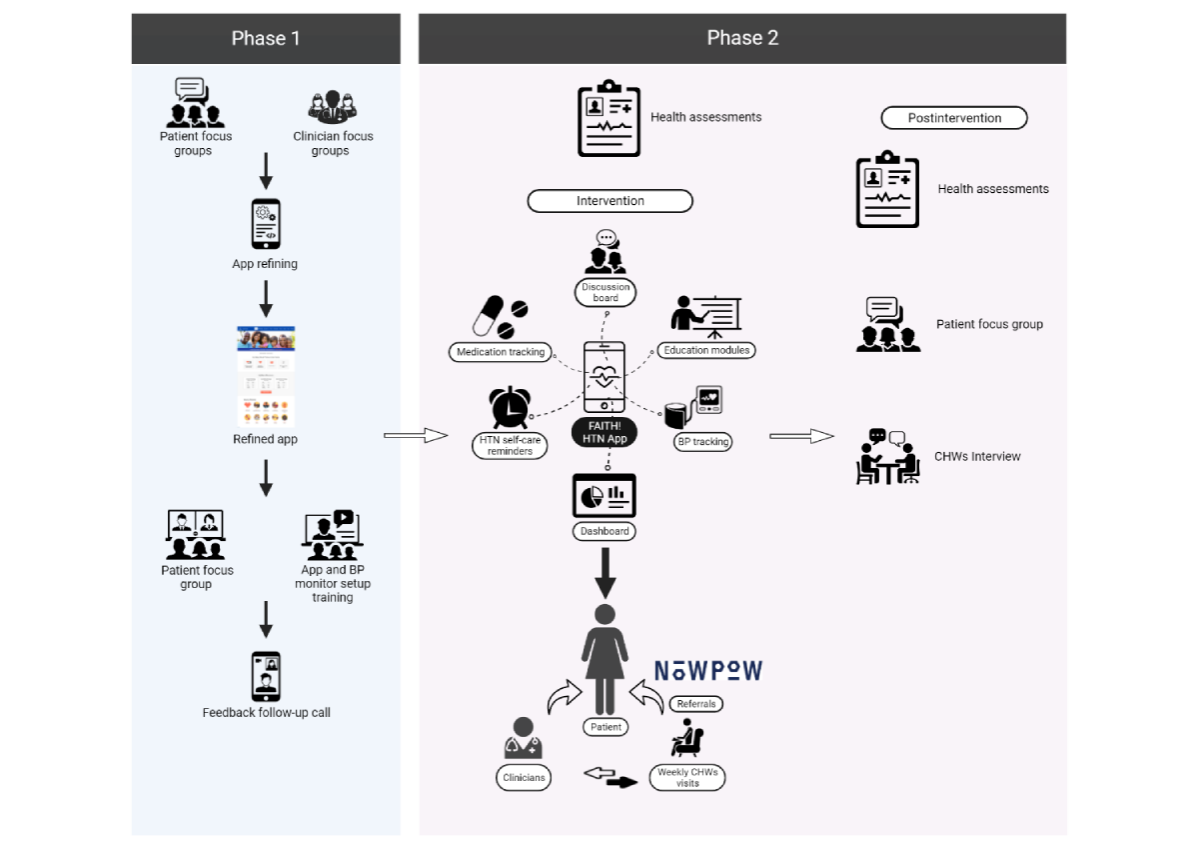
Study overview by phases. BP: blood pressure; CHW: community health worker; FAITH!: Fostering African-American Improvement in Total Health!; HTN: hypertension.

The Fostering African-American Improvement in Total Health! (FAITH!) program at the Mayo Clinic is a CBPR partnership focused on CVH promotion among African American patients [38,39]. FAITH! collaborated with the Minnesota Department of Health Cardiovascular Health Unit and 2 FQHCs in the Minneapolis-St Paul, Minnesota metropolitan area (NorthPoint Health and Wellness Center and Open Cities Health Center), to address the mutually identified priority of uncontrolled hypertension among African American patients. Given the success and effectiveness of a FAITH!-led mHealth cardiovascular intervention (FAITH! App) among participants of a similar demographic [40], the Minnesota Department of Health and FQHCs sought to integrate the intervention into clinical and community settings to improve hypertension self-management among African American patients with uncontrolled BP. The study used an integrated care model [41-43] with CHW support to deliver health education and SDOH referrals (using NowPow, a web-based personalized community referral platform) and promote patient engagement with a smartphone-based app and wireless Bluetooth-enabled BP monitors. Using a CBPR approach, the mHealth FAITH! App was refined based on the feedback from the clinicians and African American patients seeking care at the 2 FQHCs. The study had two phases: (1) a qualitative evaluation for refining the intervention and (2) a single-arm pre-post pilot test of the intervention. An overview of the study phases is shown in Figure 1.

### Study and Intervention Theoretical Framework

**Figure 2:**
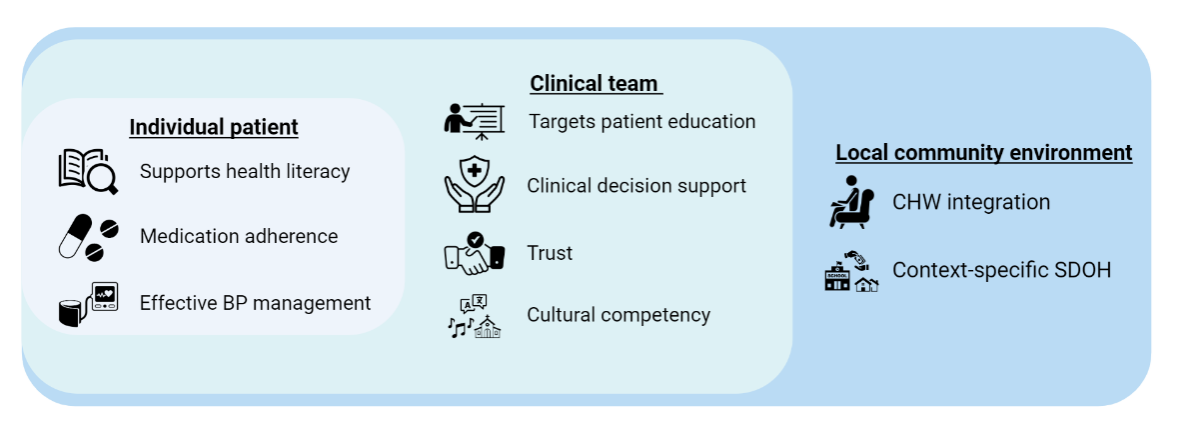
Social-ecological model guiding multilevel intervention design. BP: blood pressure; CHW: community health worker; SDOH: social determinants of health.

Our study design and intervention components target several spheres of the social-ecological model [44] (Figure 2) and the Consolidated Framework for Implementation Research (CFIR) [45] to better serve African American patients with hypertension by considering the barriers to and facilitators of hypertension management for them. These include individual patient (supporting health literacy, medication adherence, and effective BP management), clinician or clinical team (targeting patient education, clinical decision support, trust, and cultural competency), and local community environment (adding CHW integration to address health disparities and context-specific SDOH).

The CFIR allows for a systematic assessment of multilevel implementation contexts to identify factors that may influence intervention implementation and effectiveness [45]. We incorporated several domains from the CFIR to further enhance the intervention development. These include the *Intervention Characteristics* domain, as our intervention was derived from an evidence-based, community-designed, and culturally tailored app for African American individuals focused on CVH promotion, which resulted in improved BP control in this population [40,46]. This also supports its design quality and adaptability. The mHealth component in tandem with support mechanisms (CHWs and clinicians) further strengthens its advantages. Furthermore, by way of the *Outer Setting* domain, the intervention addresses the patient needs and resource construct by providing an app to address hypertension management and a CHW to focus on SDOH. The *Inner Setting* domain informed the need to culturally tailor the intervention to meet structural (FQHC), community-based (African American community), and communication-related (clinicians and CHWs) demands. The *Characteristics of Individuals* domain was addressed through the integration of the health belief model [47] in the intervention to increase patients’ hypertension knowledge and self-efficacy for hypertension self-management. Finally, the *Process* domain was incorporated with the formative phase 1 that gleaned input from African American community members to refine a culturally tailored intervention and increase stakeholder engagement (ie, patients and clinicians). The intervention is implemented in clinical practice and is complementary and community based (app tool, support of CHWs, and convenient locations for participants). The reflecting and evaluating CFIR construct was built into the intervention model by way of the app features (self-monitoring and sharing board) and CHW weekly visits.

### Setting and Study Population

Both FQHCs predominantly served low-income patients with a high burden of negative SDOH, with more than half (65%) of the adult patients diagnosed with chronic hypertension and 51% of these patients having uncontrolled hypertension. In collaboration with each FQHC, the study team focused on African American patients with uncontrolled hypertension for the provision of the mHealth intervention.

### Phase 1

#### Participant Screening and Eligibility Criteria

As for patients, those who received primary care at 1 of the 2 FQHCs, self-reported a medical history of hypertension, self-identified as African American, and were aged ≥18 years were eligible for phase 1. As for clinicians (physicians, registered nurses, physician assistants, etc), those who were employed and actively providing care at 1 of the 2 FQHCs were eligible for phase 1. Exclusion criteria included the inability to commit to the 2 focus groups conducted before and after refining the app and diagnosis of a serious medical condition or disability that would make participation difficult (ie, visual or hearing impairment or a mental disability that would preclude the independent use of the app).

#### Recruitment

Patients from the partnering FQHCs were recruited over 2 weeks through promotion by the study staff and FQHC leadership, referrals by the FQHC clinicians or staff, and flyers posted in the clinics. All interested patients received recruitment materials containing a brief description of the study, key contact information of the Mayo Clinic project coordinator, and study inclusion and exclusion criteria (Multimedia Appendix 1). Clinicians from the partnering FQHCs were recruited to participate in the focus groups through in-person promotion by the FQHC leadership and flyers posted in the clinics. Upon confirmation of their interest and eligibility by the Mayo Clinic project coordinator, participants (both patients and clinicians) provided verbal consent via telephone or in person before participating in the first focus group session.

#### Data Collection and Outcomes: App Refinement and Beta Testing

This study phase was implemented at both FQHCs from January 2020 to March 2020. The study team conducted a total of 4 in-person focus groups with patients (2 focus group sessions per FQHC) and 2 in-person focus groups with clinicians (1 focus group session per FQHC) to review the existing FAITH! App and proposed intervention components. The focus group sessions were cofacilitated by a trained qualitative research moderator in collaboration with the primary study team. Each focus group session was audio recorded and approximately 1 hour in duration. The purpose of the patient focus groups was to receive feedback on the patients’ app experience and its content for further refinement. Clinician focus groups were conducted to (1) assess the modifications needed to the mHealth app to address the needs of patients within their practice and (2) receive input on the outpatient workflow of each clinical practice for the management of patients with hypertension.

Each patient participated in 2 sessions. During the first focus group session, patients were given access to the app either by downloading the app to their personal smartphones or via a study-provided loaner tablet device. Patients received basic training on the app features and were allowed to continue using the app for 10 weeks. Feedback from the first focus groups was synthesized by the research team and provided to the app developers to tailor and refine the existing FAITH! App to ensure optimal user app engagement and usability. The second focus group session was held via the web approximately 2 months later using a videoconferencing platform owing to the COVID-19 pandemic. At this session, the study team informed patients of the app modifications based on their prior feedback, assessed their impressions of the modifications, and reviewed their experience using the refined FAITH! App. Furthermore, patients were instructed on how to set up a home, Bluetooth-enabled, and wireless BP monitor (Omron Evolv, Omron Healthcare, Inc) for use with the app [48]. Following the focus groups, the BP monitors were directly mailed to patients to test its automatic synchronization with the refined FAITH! App. Two weeks later, the study team followed up with each patient through telephone interviews to gather any additional feedback on the BP monitor and the app overall. Feedback from the interviews was used to adjust and streamline the app and BP monitor setup process through the creation of a user-friendly manual for use in the implementation phase (phase 2).

#### Measures: Primary Outcome

The primary outcome of this phase was completing refinements to the app by incorporating inputs from patients on how to most effectively support hypertension self-management and from clinicians on how the app could enhance patient care.

#### Data Analyses

To refine the FAITH! App in phase 1, discussions from the first cycle of patient and provider focus groups were audio recorded and transcribed. Immediately after the first cycle, a summary analysis of the transcribed feedback from the discussions was conducted by the moderator (MC). Key themes from the transcripts were extracted to be relayed to the app developer (CareHubs, Inc), who refined the app as necessary. After the suggested app revisions were completed, a second cycle of focus groups was conducted with the original focus group participants to confirm that the app has been appropriately tailored. Subsequently, the transcripts of each session were independently reviewed by 2 study team members (LB and MP) for the confirmation of summary analysis themes and the extraction of additional emergent themes. Each team member aggregated a synthesis of the major themes of participant feedback by interview questions and suggested revisions. The initial thematic analysis incorporated codes from the Health-Information Technology Usability Evaluation Model [49]. A third team member (ML) assisted with discrepancy resolution to ensure consensus. Transcripts from the phase-2 patient and CHW postintervention evaluation on the overall appraisal of and satisfaction with the intervention were evaluated through content analysis. The data were organized using Microsoft Excel (Microsoft Corp), and content analyses were performed using the NVivo software (version 10, QSR International).

### Phase 2

#### Participant Screening and Eligibility Criteria

To be eligible for phase 2, in addition to meeting the eligibility criteria from phase 1, patients had to meet the following criteria: a documented diagnosis of hypertension in the electronic medical record (EMR), uncontrolled hypertension (defined as BP ≥140/90 mm Hg [or the threshold at which pharmacological treatment should be initiated according to American College of Cardiology/American Heart Association guidelines]) [50] at the most recent outpatient evaluation (within the last year, with or without prescribed BP medications), at least 1 visit to the FQHC in the prior year, ownership of a personal smartphone with an iOS (Apple, Inc) or Android system, access to the internet on at least a weekly basis, an active email address, and basic internet navigation skills (eg, ability to search for information on websites and open and send emails). Exclusion criteria included the diagnosis of a serious medical condition or disability that would make participation difficult (ie, visual or hearing impairment or a mental disability that would preclude the independent use of the app) and the absence of a primary care clinician at the partnering FQHC.

#### Recruitment

Patients were recruited from 1 partnering FQHC (NorthPoint), as the other FQHC (Open Cities) partner was unable to continue study recruitment because of the impact of the COVID-19 pandemic on their clinical practice. The study team and Open Cities remained engaged with other FAITH! program–led response efforts (eg, community COVID-19 testing) [51]. Recruitment occurred during a 3-month period. Clinicians referred potentially eligible African American patients with uncontrolled hypertension to the study team. All interested patients received recruitment materials (via email or as a hard copy) containing a brief description of the study, key study contact information of the Mayo Clinic project coordinator, and the study inclusion and exclusion criteria (Multimedia Appendix 1). The Mayo Clinic project coordinator then contacted patients via telephone to verify their interest in participation and eligibility criteria. In addition, patients and clinicians received information on the study objective and components through a web-based informational session (via a web-based meeting platform) held by the study staff. Once the patients’ interest and eligibility were confirmed, the project coordinator scheduled in-person health assessments to collect written informed consent and study measures.

#### Intervention: Refined FAITH! Hypertension App and CHW-Enhanced Intervention

##### The FAITH! Hypertension App

The patients followed the app-based intervention for 10 weeks (May-July 2021). The culturally tailored intervention consisted of a 10-module educational series focused on hypertension and cardiovascular risk factors with multimedia features, including interactive self-assessments, medication, BP self-monitoring, and social networking. Patients were encouraged to follow each module on a weekly basis (eg, *Week 1: Intro to CV Risk Factors* and *Week 2: High BP*). Patients were provided with a home Omron Evolv BP monitoring system to allow for the self-tracking of BP measurements. Patients were encouraged to track their BP measurements for at least 3 days weekly. The app included pre-post module self-assessments (quizzes) and a tracking component for patients to input their medications. The self-monitored BP measures were automatically synchronized with the app. The app sharing board was a moderated discussion platform and feed for patient interaction by posting healthy lifestyle practices through text, photographs, and videos to foster discussion on hypertension management barriers and facilitators. The project team posted weekly on the sharing board on hypertension-related topics to generate discussion among patients. Patients maintained app access via their personal smartphone for the duration of the study.

##### CHW Visits

In total, 2 African American CHWs were hired as a part of the study team to provide support to the enrolled patients from the participating FQHC. In conjunction with the use of the FAITH! Hypertension App, patients met with the same CHW weekly (predominantly remotely via telephone or Zoom [Zoom Video Communications, Inc]) as per their preference over the 10-week intervention to monitor their progress. During these visits, the CHWs reviewed patients’ average BP and patient-tracked medication adherence. In addition, CHWs monitored app engagement, including progress through the app educational modules and the use of the sharing board (intervention receipt); medication adherence; and BP self-monitoring via an app dashboard summary (intervention enactment and behavioral skills). CHWs also reviewed patients’ SDOH, including barriers to healthy lifestyle and hypertension management (eg, inadequate access to food, housing, and medications), and the services provided (eg, medication and food vouchers). All visit proceedings were recorded on the digital *Activities Form* based on patient feedback and the expressed needs, and the CHWs leveraged the NowPow platform to refer them to appropriate community resources. NowPow is a digital platform that links patients with chronic health conditions in underresourced communities to a full spectrum of vital community-based health and social services, from basic needs (eg, food, housing, and financial assistance) to more specialized services (eg, mental health counseling and caregiver support).

At the end of each week, CHWs collected the completed *Activities Form* and securely transferred them electronically to FQHC staff to upload them to each patient’s EMR for review by their clinicians.

##### CHW Training

The CHWs received training from the study’s principal investigator (PI) on the FAITH! Hypertension App features and reviewed all content, particularly the 10 CVH-focused education modules. In addition, the CHWs received web-based training for 2 months via the Association of Black Cardiologists, Inc, Community Health Advocate Training (CHAT) program [52] using the evidence-based National Institutes of Health (NIH)/National Heart, Lung, and Blood Institute (NHLBI) training manual, *With Every Heartbeat is Life, A Community Health Worker’s Manual for African Americans* [53]. Each CHW received a CHAT program certificate of completion for proficiency in providing CVH education and promotion to underserved African American patients with hypertension. In tandem (and concurrently) with the CHAT program, the study PI also trained the CHWs using the NIH/NHLBI training manual, which was also provided to the CHWs as a supportive patient health education tool, with an emphasis on the hypertension curriculum. The accompanying NIH/NHLBI patient manual, *On the Move to Better Heart Health for African Americans*, was also incorporated into the FAITH! Hypertension App to further reinforce CVH promotion among patients [54]. The CHWs were provided with a tablet device that included the FAITH! Hypertension App and digital *Activities Form* for use during weekly patient visits. To provide comprehensive information on the activities conducted during weekly patient visits to the study team and partnering clinic, CHWs were trained on the proper completion of the weekly visit *Activities Form*. The forms included a checklist of standardized procedures for completion during the weekly visits (eg, review of BP measurements, medications, app education modules, and SDOH; Multimedia Appendix 1). Finally, CHWs received training on the use of the NowPow platform to issue appropriate referrals for community resources to patients to address their social needs based on a review of their SDOH.

The fidelity of intervention delivery and protocol adherence was assessed through the random monitoring of the recordings of patient-CHW encounters and qualitative patient interviews by the study PI, with feedback provided to CHWs for quality improvement.

#### Data Collection and Outcomes: Baseline and Postintervention Assessments

**Figure 3:**
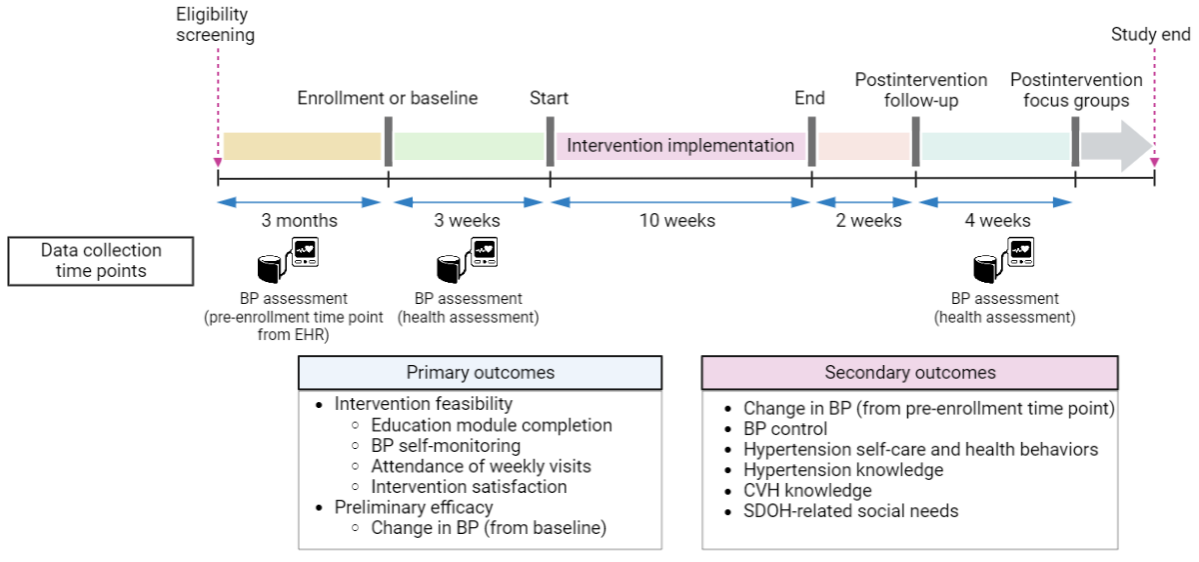
Study data collection timeline. BP: blood pressure; CVH: cardiovascular health; EHR: electronic health record; SDOH: social determinants of health.

All patients completed the written consent form with the study coordinator at scheduled health assessments at the FQHC clinical site. After confirming patient enrollment, the project CHWs led health assessments to collect clinical data and complete electronic surveys at baseline and after 10 weeks of intervention. The clinical data included BP measurements (average BP of 3 readings in a sitting position, measured using an oscillometric automated device [Omron Evolv]), weight (measured using a digital scale), and height (measured using a stadiometer); proper social distancing precautions, masking, and hand sanitization in accordance with the Centers for Disease Control and Prevention guidelines during the COVID-19 pandemic were followed while collecting the data. In addition, the study participants were asked to complete an electronic survey to evaluate their baseline level of health knowledge and health service use, medication adherence, hypertension knowledge, hypertension self-care activities, health behaviors, and SDOH. Similar procedures were followed to collect immediate postintervention clinical data and survey data. Figure 3 provides details of the data collection timeline and outcome measures (with a focus on the BP collection time points).

A web-based focus group was held with patients after the intervention to assess their experience and satisfaction with the FAITH! intervention as a whole and the FAITH! Hypertension App–specific components (refer to Multimedia Appendix 1 for the moderator guide). In addition, a group interview with the 2 FAITH! program CHWs was conducted to evaluate their experience and satisfaction with the FAITH! Hypertension App.

#### Measures

##### Primary Outcomes

The primary outcomes were intervention feasibility (engagement and satisfaction) and preliminary efficacy (change in BP from baseline to the immediate postintervention assessment).

###### Feasibility

Intervention engagement was assessed through app education module completion (the percentage of education modules completed per patient), BP self-monitoring (mean number of weekly BP measurements per patient), and the attendance of weekly visits (mean number of completed weekly visits per patient). Intervention satisfaction was assessed quantitatively via survey measures (eg, acceptability, app rating, and CHW interaction) and qualitatively via the postintervention focus group. Acceptability was assessed through 1-item: “I would recommend this program to other patients/family/friends” (response options: “yes” and “no”). Patients were asked to rate the app overall in assisting them with hypertension self-management (on a scale ranging from 1 to 10). Rating options for the app features and CHW interaction were “fair,” “good,” “very good,” and “excellent.” Moreover, specific to app satisfaction, the usability of the FAITH! Hypertension App was evaluated at the postintervention assessment using the Health Information Technology Usability Evaluation Scale (Health-ITUES) [55], a customizable mHealth intervention usability assessment instrument. The 20-item scale includes 4 subscales: impact (3 items), perceived usefulness (9 items), ease of use (5 items), and user control (3 items). Each item is rated on a 5-point scale ranging from 1 (strongly disagree) to 5 (strongly agree), with higher scale values indicating greater perceived usability of the intervention. The overall Health-ITUES score was calculated as the mean of all 20 items, with each item being equally weighted. Similar to the overall score, the score of each subscale was calculated as the mean of its items.

###### Preliminary Efficacy

The primary outcome was the absolute change in BP (both systolic and diastolic BP [mm Hg]) from baseline to the immediate postintervention assessment (at the end of the 10-week intervention).

##### Secondary Outcomes

###### Change in BP (From the Pre-enrollment Time Point) and BP Control

The absolute change in BP (both systolic and diastolic BP [mm Hg]) from the pre-enrollment to immediate postintervention (at the end of the 10-week intervention) time points was measured as a secondary outcome. The pre-enrollment BP was obtained from the most recent ambulatory clinic encounter recorded in the EMR, whereas the immediate postintervention BP was obtained through direct measurement at the health assessments. Absolute change in BP control was defined as a target BP of <140/90 mm Hg. The proportion of patients who reached the target BP before and after the intervention was assessed.

###### Hypertension Self-care and Health Behaviors

The Hypertension Self-Care Activity Level Effects (H-SCALE) questionnaire was administered to assess levels of self-care [56]. The 31-item instrument assesses 6 hypertension behavioral self-care activities recommended for optimal hypertension management, namely BP medication adherence, the adoption of a low-salt diet, regular PA for ≥30 minutes per day, smoking cessation, weight maintenance, and limiting alcohol intake. The instrument demonstrated reliability and validity in African American patients with hypertension receiving care at an FQHC and was superior to alternative measures of hypertension self-care [56,57]. To further assess diet, self-reported daily fruit and vegetable intake was assessed using 2 items adapted from previously developed instruments assessing fruit and vegetable intake [58-60]. Respondents reported daily servings of fruits and vegetables consumed. This instrument was previously validated in a similar population of African American individuals [61]. In total, four intensity levels of PA from a 7-day recall were assessed using the short form of the International PA Questionnaire survey [62]: (1) vigorous-intensity activity, such as aerobics; (2) moderate-intensity activity, such as leisure cycling; (3) walking; and (4) sitting. The International PA Questionnaire has also been validated among African American individuals [63].

###### Hypertension and CVH Knowledge

Individual hypertension knowledge was assessed using an 11-item questionnaire that was previously validated in patients with hypertension in primary care clinics [64]. One point was allocated for each correct answer to generate a summary score ranging from 0 to 11. To assess patients’ understanding of the education module content, CVH knowledge was measured using premodule and postmodule self-assessment quizzes within the app modules (percentage of questions answered correctly).

###### SDOH-Related Social Needs

Use metrics were also collected on the NowPow platform (eg, the number of SDOH screenings completed, specific patient-identified needs, and number and type of referrals shared with patients for community-based resources and services). The Protocol for Responding to and Assessing Patients’ Assets, Risks, and Experiences (PRAPARE) assessment tool was used to assess SDOH for descriptive baseline sociodemographics [65].

#### Data Analyses

Descriptive statistics of the primary and secondary measures were compared between the baseline and postintervention time points. Categorical variables were expressed as percentages, whereas continuous variables were expressed as mean (SD). Furthermore, B*P* values between the baseline and postintervention time points were compared using paired 2-sided *t* tests (primary outcome). Paired *t* tests were also used to compare pre-enrollment and postintervention B*P* values (secondary outcome). All statistical analyses were performed using SAS (version 9.4, SAS Institute Inc).

### Ethics Approval, Informed Consent, and Participation

The study was approved by the Mayo Clinic Institutional Review Board and registered (ClinicalTrials.gov NCT04554147). Verbal (phase 1) or written (phase 2) informed consent was obtained from all participants before their participation in the study. Participants received incentives during both phases of the study. During phase 1, patient participants received a total of US $50 by cash card, US $25 at the first focus group and US $25 at the second focus group. Patient participants were also given the wireless BP monitor as an incentive. Clinicians received compensation through the meals provided at each focus group. During phase 2, patients received US $50 at enrollment, US $25 at the immediate postintervention health assessment, and US $50 at the postintervention focus group. Participants also received the Omron Evolv BP monitor as an incentive at the baseline health assessment. The data were deidentified to ensure privacy and confidentiality.

## Results

### Phase 1 App Refinement and Beta Testing

A total of 13 African American patients and 16 clinicians from Open Cities (7/16, 44%) and NorthPoint (9/16, 56%) participated in the focus group series to provide insights on the integration of the FAITH! mHealth intervention enhanced with CHW support into a community or clinic-based setting. The patient demographics were as follows: 77% (10/13) were women, 69% (9/13) were aged >50 years, 75% (9/13) reported at least some college or higher education, and 46% (6/13) reported an annual household income of <US $50,000. More than half (8/13, 67%) of the patients reported being comfortable using mobile technology, with 69% (9/13) using a smartphone or a tablet. Clinician demographics were as follows: 88% (14/16) were women, 31% (5/16) were physicians, and 50% (8/16) were aged >50 years. Clinicians were also comfortable with mobile technology (13/16, 81%) and used mobile technologies (16/16, 100% reported the use of smartphones and 10/16, 63% reported the use of tablets).

**Figure 4:**
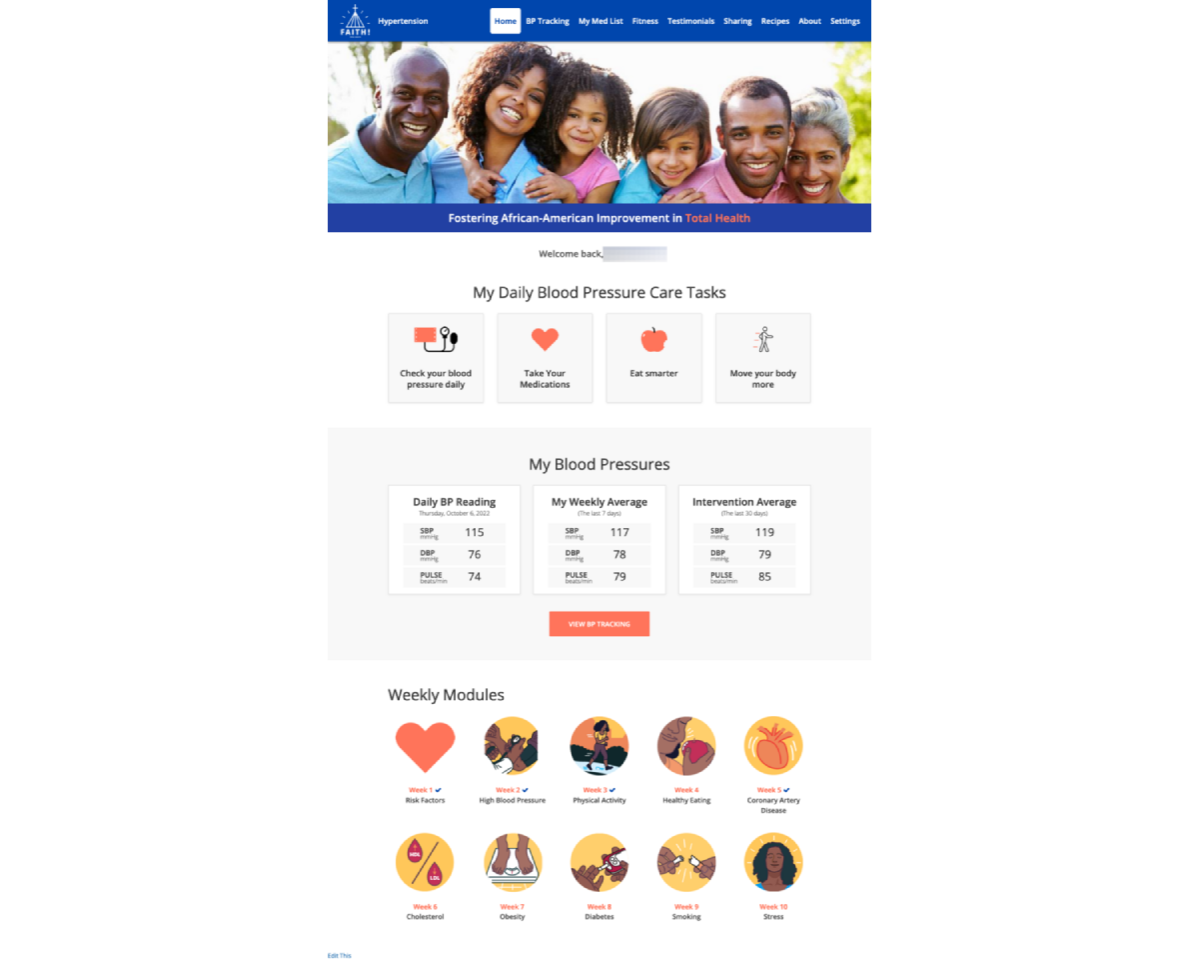
FAITH! (Fostering African-American Improvement in Total Health!) Hypertension App home page. DBP: diastolic blood pressure; SBP: systolic blood pressure.

On the basis of the suggestions from both groups, several modifications were made to the existing FAITH! App (Table 1). Key changes were focused on patient-centeredness to promote hypertension self-management, including the addition of a BP dashboard with automatic synchronization with an Omron Evolv BP monitor for BP tracking, a medication tracking feature, care task reminders, and fitness videos to support regular PA (Figure 4). Clinicians indicated that the app-based intervention would place patients in the “driver’s seat” of their own hypertension management for accountability. They also noted that CHWs would enhance the intervention and their clinical practice by addressing additional needs beyond BP control, such as the SDOH. At the regroup focus group with patients for receiving further feedback on the app refinements, patients indicated overall positive impressions of the app and additional features. Furthermore, there was a collective agreement to rename the app “FAITH! Hypertension App.” After a 2-week beta testing of the app with the provided BP monitor, patients indicated that the synchronization of the monitor with the app was a major strength, convenient, and supportive of hypertension management. However, requests were relayed to improve the FAITH! Hypertension App and Omron app download processes for future patients in the pilot study.

**Table 1:** Phase 1 app refinement: patient suggestions and study team modifications.

Suggested modifications	Modifications completed
Blood pressure tracking feature	Blood pressure tracking tab with the display of blood pressure measurements from directly synced wireless blood pressure monitor introduced Dashboard to display daily blood pressure, average weekly blood pressure, and average blood pressure throughout intervention Color coding for the deciphering of normal versus high blood pressure ranges
Medication tracking feature	My Med List tracking tab with options to enter medications (name, dose, route, and frequency) introduced
Sociocultural context updates: content on racism and its impact on hypertension control and messaging with an emphasis on stress management with the integration of personal faith	Updated the education modules to include content on unique stressors experienced by African American individuals, including racism or discrimination and their impact on health Techniques, including mindfulness and religious and spiritual coping, incorporated into stress management education module
Alerts or reminders for hypertension self-care	Care task reminders for hypertension self-management added to the home page. Reminders include checking blood pressure daily, adhering to medication, eating smarter, and moving or getting regular physical activity To support regular physical activity, a Fitness tab was added with 6 fitness videos led by a Mayo Clinic fitness instructor. Workouts included warm-up, cardio-aerobics, stretching or flexibility exercises, and cooldown
General app content updates	Enlarged the FAITH!^a^ logo and moved its placement to increase readability Included the FAITH! Hypertension App name on the home page Education module icons were culturally tailored to be more reflective of the African American community as a means of expressing the study team’s cultural humility

^a^FAITH!: Fostering African-American Improvement in Total Health!

### Phase 2 Pilot Study

#### Study Participant Characteristics

**Figure 5:**
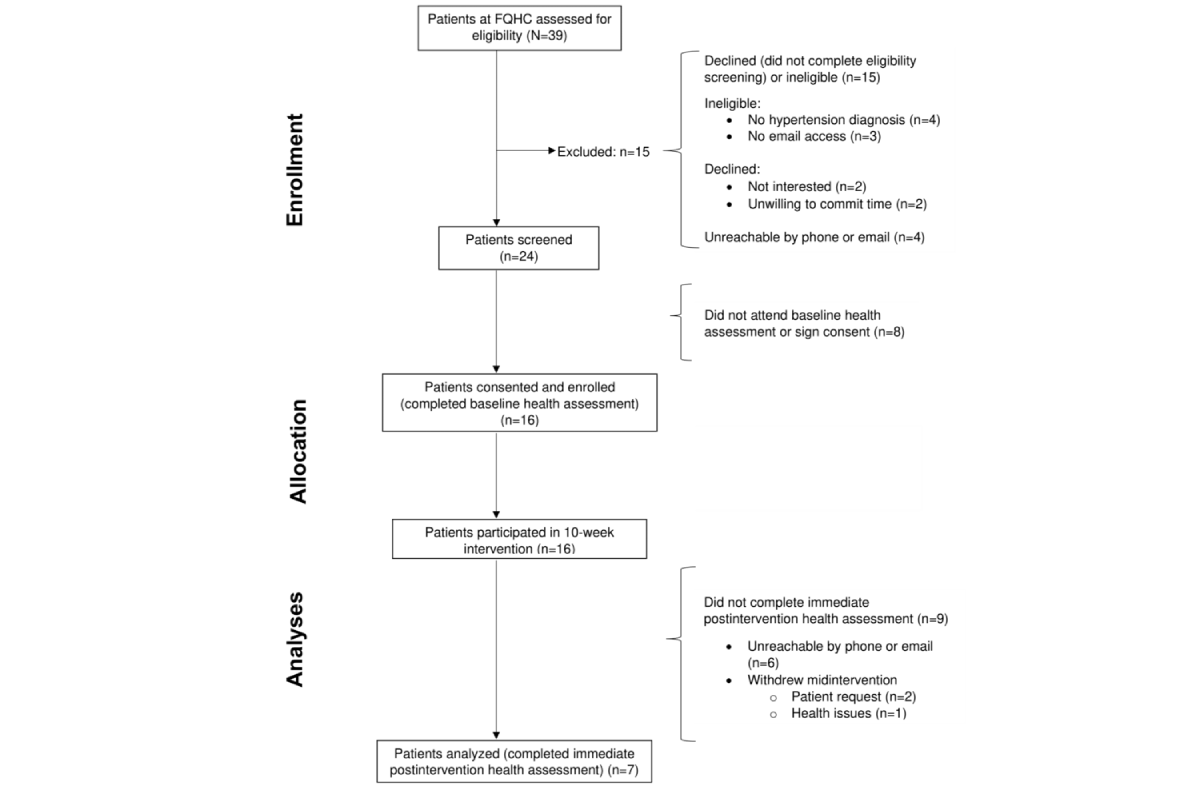
Modified CONSORT (Consolidated Standards of Reporting Trials) flow diagram: screening, enrollment, and follow-up of participants. FQHC: federally qualified health center.

Sample characteristics are listed in Table 2. Patients were predominantly women (12/16, 75%), with a mean age of 52.6 (SD 12.3) years. All enrolled patients (16/16, 100%) completed the baseline health assessment, of whom 44% (7/16) completed the immediate intervention health assessment and made up the final analytic cohort (for baseline vs postintervention comparisons; refer to Figure 5 for a modified CONSORT [Consolidated Standards of Reporting Trials] flow diagram). At enrollment, the mean systolic BP and diastolic BP of the eligible patients were 146.3 (SD 17.1) mm Hg and 94.4 (12.6) mm Hg, respectively. Overall, the cohort had a mean BMI of 37.4 (SD 12.0) kg/m^2^, and all patients (16/16, 100%) indicated a diagnosis of at least 1 chronic medical condition (diabetes, hyperlipidemia, obesity, or tobacco dependence) in addition to hypertension. Patients had an overall high burden of negative SDOH, with the majority reporting an annual household income <200% of the federal poverty level and high stress levels. All patients owned a personal smartphone (16/16, 100%), and most of them (14/16, 88%) had internet access beyond their smartphone. Patients reported overall comfortability with mobile technologies (11/16, 69%) and confidence in using the internet to make health-related decisions (14/16, 88%).

**Table 2:** Patient baseline characteristics (phase 2; n=16).

Characteristics				Value^a^
Age (years), mean (SD)				52.6 (12.3)
Gender, women, n (%)				12 (75)
**Clinical characteristics**				
	Systolic BP^b^ (mm Hg), mean (SD)^c^			146.3 (17.1)
	Diastolic BP (mm Hg), mean (SD)^c^			94.4 (12.6)
	BMI (kg/m^2^), mean (SD)			37.4 (12.0)
	Hypertension diagnosis and chronic condition, n (%)			16 (100)
**Social determinants of health^d^, n (%)**				
	Annual household income <200% of the federal poverty level (n=6)			4 (67)
	Has housing			9 (64)
	Worried about losing housing			3 (38)
	Less than high school degree			1 (7)
	Part-time or full-time employment (n=14)			3 (21)
	Medicaid or Medicare insurance (n=13)			9 (69)
	**Unmet health-related social need over past year**			
		Food	5 (63)	
		Utilities	5 (56)	
		Clothing	4 (50)	
	Lack of transportation			3 (19)
	Social integration <5 times per week (n=13)			7 (54)
	Stress			11 (85)
	Physical or emotional safety at home (n=12)			7 (58)
**Digital determinants of health^e^, n (%)**				
	Smartphone ownership			16 (100)
	Internet access beyond smartphone			14 (88)
	Comfortable with mobile technology			11 (69)
	Confidence using internet to make health decisions			14 (88)

^a^Includes all patients enrolled at the baseline. Values are indicative of complete and valid data. Incomplete data were not included in the analyses.

^b^BP: blood pressure.

^c^Note that blood pressure was last recorded within patient electronic medical record at the time of eligibility screening.

^d^Adapted from the Protocol for Responding to and Assessing Patients’ Assets, Risks, and Experiences (PRAPARE) tool [65,66].

^e^Questions developed by the study team based on the domains outlined by Richardson et al [65,66].

#### Feasibility

##### Engagement

As for the enrolled patients’ engagement with the app, 4 (25%) of the 16 patients completed all 10 education modules (including a total of 20 premodule and postmodule quizzes), whereas 38% (6/16) completed at least half of the 10 modules (Multimedia Appendix 1). The most commonly completed modules (each completed by 6/16, 38% enrolled patients) were on the following topics: *Risk Factors*, *High BP*, *PA*, and *Coronary Artery Disease*. There were no substantial differences in the completion of the education modules between the participants in the final sample and the entire sample of 16 participants enrolled in the study. As for BP self-monitoring, 10 (63%) of the 16 patients obtained a mean of 42 (SD 53) BP measurements throughout the intervention, equating to an average of 4 measurements per week. The CHWs followed a total of 13 patients weekly (6-7 patients per CHW). A total of 80 CHW visits were completed throughout the intervention (approximately 1 visit per patient each week).

##### Satisfaction

The salient themes and illustrative quotes associated with patients’ satisfaction with the intervention are presented in Table 3. There was high acceptability of the intervention, as all 7 patients indicated that they would recommend the FAITH! Hypertension App to other patients. Furthermore, patients gave the app a rating of 9 (out of 10) on its helpfulness in managing BP. All FAITH! Hypertension App features received a “Very Good” to “Excellent” rating from >70% of the patients (Multimedia Appendix 1). There was a unanimous “Excellent” rating for the study CHWs from all patients. As for app usability, the app received a mean total Health-ITUES score of 4.1 (SD 1.4) from patients. The mean Health-ITUES subscale scores were overall moderately high: impact, 4.3 (SD 1.5); perceived usefulness, 4.2 (SD 1.4); perceived ease of use, 4.0 (SD 1.4); and user control, 3.9 (SD 1.4). From their standpoint, both CHWs indicated that patients found the app interesting with useful content and helpful for hypertension self-management. CHWs found the intensive, multisource training useful, as they felt well prepared to partner with their patients on hypertension self-management. Similar to the patients, the CHWs reported a disconnect between the FAITH! program team and FQHC staff and suggested more integration with a consistent point of contact.

**Table 3:** Patients’ satisfaction with the Fostering African-American Improvement in Total Health! (FAITH!) Hypertension App and community health workers (CHWs): salient themes with illustrative quotes.

Themes		Illustrative quotes
**Experience with the FAITH! Hypertension App**		
	Informative	“Surprised by the things I didn’t know that I assumed I knew and found out I didn’t have a clue.”
	Engaging	“It makes you pay attention. I’m 64. Been eating right and exercising, maintaining weight since I found out I was diabetic. But the smoking is a hard habit to break. Program [FAITH!] keeps you on your toes.”
	Increased awareness and confidence	“A little more confident. Made me feel good to know that something I was doing was actually working to keep my health in shape.”
	Easy to use	“I liked how the modules worked where it automatically let you click the next button without having to search. Easily put you into the next week. Quite easy to navigate.”
	Importance of peer support	“We were able to go on the message board or read other stuff that other people were going through. See that you’re not the only one going through certain things.”
**Experience with the CHWs**		
	Encouraging	“Need a lot of accountability and times I wanted to give up—I’m done with this; I have other stuff going on. But she was always there to encourage me to finish the program and I’m glad I did.”
	Supportive	“[CHW] always would answer any question. I gave up and wanted to give my BP monitor back and she called me back the next week and was so helpful.”

#### Preliminary Efficacy: Change in BP

At the baseline health assessments, the mean systolic and diastolic BP were 149.7 (SD 15.3) and 93.4 (SD 8.9) mm Hg, respectively (Table 4). From the baseline to immediate postintervention time points, there were reductions in both mean systolic (−6.5 mm Hg; *P*=.15) and mean diastolic (−2.8 mm Hg; *P*=.78) BP, although these differences were not statistically significant. The absolute change in systolic BP (mm Hg) from the pre-enrollment time point (extraction from the most recent clinic visit) to the immediate postintervention time point was −11.4 (SD 17.6) mm Hg, which was nonsignificant (*P*=.14). The proportion of patients with controlled BP increased from 0% at the baseline time point to 29% at the immediate postintervention time point (*P*=.17).

**Table 4:** Primary and secondary outcomes at the baseline and immediate postintervention time points^a^.

Characteristics			Baseline (n=7)	Postintervention assessment (n=7)	*P* value
**BP^b^**					
	Systolic BP (mm Hg), mean (SD)		149.7 (15.3)	143.2 (17.0)	.15
	Diastolic BP (mm Hg), mean (SD)		93.4 (8.9)	90.6 (19.9)	.78
	Controlled BP (<140/90 mm Hg), n (%)		0 (0)	2 (29)	.17
**Hypertension self-care activities**					
	**H-SCALE^c^,^d^ subscale, mean (SD)**				
		Medication adherence (n=6)	19.0 (1.4)	18.1 (2.7)	.22
		Low-salt diet adherence (n=4)	49.5 (3.0)	40.6 (12.3)	.88
		Physical activity adherence (n=6)	5.3 (4.5)	8.4 (3.6)	.09
		Nonsmoking adherence (n=6)	4.5 (6.3)	3.9 (5.4)	.99
		Weight management adherence (n=6)	34.7 (7.3)	35.3 (6.0)	.76
		Alcohol abstinence (n=7)	0.4 (0.8)	3.7 (2.5)	.16
	**Health behaviors^e^, mean (SD)**				
		Fruit and vegetable intake (servings per day)	4.6 (2.8)	5.4 (3.0)	.25
		Vigorous physical activity (days per week)	1.8 (1.7)	1.3 (1.6)	.35
		Vigorous physical activity (minutes per day)	33.3 (23.1)	75.0 (63.6)	.39
		Moderate physical activity (days per week)	1.9 (1.7)	2.7 (1.9)	.32
		Moderate physical activity (minutes per day)	24.0 (7.1)	67.5 (75)	.42
		Walking (days per week)	1.7 (1.5)	5.7 (1.9)	.004
		Walking (minutes per day)	22.5 (9.6)	77 (70)	.41
		Sitting (hours per day)	7.8 (6.1)	9.5 (9.5)	.43
		Sitting (minutes per day)	25.0 (22.9)	28.3 (2.9)	.30
Hypertension knowledge total score^f^, mean (SD)			9.9 (0.9)	9.4 (1.6)	.51
**Cardiovascular health knowledge, mean percentage correct^g^, mean (SD)**					
	Risk factors		55.7 (17.6)	83.5 (18.1)	.004
	High BP		50.0 (18.6)	100.0 (0.0)	.001
	Physical activity		72.5 (13.5)	83.3 (25.8)	.34
	Healthy eating		89.0 (17.0)	100.0 (0.0)	.17
	Coronary artery disease		66.8 (21.2)	83.3 (25.8)	.11
	Cholesterol		100.0 (0.0)	66.7 (25.8)	.03
	Obesity		93.4 (14.8)	80.0 (29.9)	.46
	Diabetes		87.5 (25.0)	100.0 (0.0)	.39
	Smoking		100.0 (0.0)	100.0 (0.0)	.99
	Stress		66.8 (27.4)	100.0 (0.0)	.09

^a^Paired *t* test calculated only for variables with sufficient data at both time points. A specific number of patients were listed if missing data for certain variables.

^b^BP: blood pressure.

^c^H-SCALE: Hypertension Self-Care Activity Level Effects.

^d^Hypertension Self-Care Activity Level Effects (H-SCALE) rubric used with permission from the developer [56].

^e^Adapted National Cancer Institute fruit and vegetable intake screener used [58-60]. Short form of the International Physical Activity Questionnaire used for physical activity measures [62].

^f^Hypertension knowledge assessed using questionnaire from the study by Abu et al [64].

^g^Mean score out of 100.

#### Hypertension Self-care and Health Behaviors

There were no statistically significant changes in the overall H-SCALE score or subscale scores (all *P*>.05; Table 4) over the course of the study. However, there were favorable trends in specific health behaviors. Combined fruit and vegetable intake increased from 4.6 (SD 2.8) to 5.4 (SD 3.0) servings per day from the baseline to immediate postintervention time points. Most forms of self-reported PA improved from the baseline to immediate postintervention time points, with the number of days of walking being the only PA that achieved statistical significance (mean 1.7, SD 1.5 vs mean 5.7, SD 1.9 days; *P*=.004). By contrast, the time spent sitting increased (both hours and minutes) from the baseline to immediate postintervention time points (*P*=.43 and *P*=.30, respectively).

#### Hypertension and CVH Knowledge

The overall hypertension knowledge was high at baseline (mean 9.9, SD 0.9), which was maintained at follow-up (mean 9.4, SD 1.6), with no statistically significant change (*P*=.51). By contrast, there were statistically significant improvements in CVH knowledge, as measured by self-assessments on the FAITH! Hypertension App. The mean percentage of correct answers increased for the risk factors (+27.8%; *P*=.004) and BP modules (+50%; *P*=.001) but decreased for the cholesterol module (−33.3%; *P*=.03).

#### SDOH-Related Social Needs

A total of 80 screenings were completed by both the CHWs, which identified 42 social needs. The 3 most common patient-identified social needs were housing, financial assistance, and utilities, with a total of 397 referrals issued to patients over the course of the intervention (Multimedia Appendix 1).

## Discussion

### Principal Findings

In this community-based participatory mixed methods study, we successfully refined and deployed a culturally tailored mHealth app to promote hypertension self-management among African American patients receiving care at FQHCs. Our study demonstrated that the FAITH! Hypertension App in conjunction with CHW support may support clinically meaningful improvements in BP and improve hypertension self-management among African American patients. After the completion of the intervention, patients had higher CVH literacy, specifically related to overall cardiovascular risk and hypertension. In addition, there were quantitative and qualitative patient data demonstrating high intervention engagement and positive satisfaction with the overall program components, including the adjunct support from CHWs, as they instilled accountability in hypertension self-management. As such, future research is warranted for the replication of these study findings in a larger sample of patients and FQHCs. In addition, a randomized controlled trial of a culturally tailored digital intervention with CHW support (vs without CHW support) may be useful for assessing differences in the improvement of hypertension management among African American patients.

### Comparison With Prior Work

Our study aligns with other studies using digital health interventions to improve BP control among African American patients but contributes several unique elements to the current literature. Consistent with a recent systematic review, our smartphone-based app intervention supported a clinically meaningful reduction in BP and changes in key health behaviors (diet and PA) [67]. Similar to a subgroup of studies within the review, the self-monitoring components with tailored advice provided through culturally tailored content and reinforcement by CHWs resulted in a 6 mm Hg reduction in systolic BP (compared with a nearly 3 mm Hg reduction in the review). Despite the lack of statistical significance, likely because of the small sample size, the observed reduction in BP is a key finding. A large-scale analysis of randomized clinical trials conducted among adult patients with hypertension found that at least a 5 mm Hg reduction in systolic BP reduced the risk of major cardiovascular events (stroke, heart failure, ischemic heart disease, and death from CVD) by 10% over 4 years of follow-up under BP-lowering treatment [68]. A randomized clinical trial with a larger sample size would likely have adequate power to detect statistically significant findings. A recent study used a socioculturally tailored mHealth digital platform to improve medication adherence and clinical factors (BP and hemoglobin A_1c_) among African American patients with hypertension and diabetes [69]. Although there were no between-group differences in BP, the intervention group demonstrated improvements in medication adherence and a 6.7–mm Hg greater reduction in systolic BP than the comparator group. Buis et al [70] demonstrated the feasibility and preliminary efficacy of SMS text message reminders in increasing BP medication adherence [70]. Contrary to these relatively static, unidirectional interventions, our intervention was interactive, allowing for a multimodality patient experience with a supportive community connector or health care educator to promote BP self-monitoring, multimedia education modules on CVH, and the sharing of hypertension self-care activities with other patients through social networking. Furthermore, prior studies using mHealth technologies to improve hypertension self-management have largely been conducted in relatively homogenous White populations [71]. African American patients were prioritized in our study, given the persistent hypertension disparities within this group and as a means to promote digital health equity (recently termed TechQuity) [72].

### Implications for Practice

Our study demonstrated the feasibility of integrating CHWs into the implementation of digital health interventions as an adjunct component to potentially enhance patient engagement with digital tools. Our qualitative findings noted that patients viewed their interactions with the CHWs as supportive and encouraging, which provided them with accountability for continued app use and adherence to hypertension self-management behaviors. Although a digital app could be implemented as a stand-alone tool to improve hypertension management in this patient population [40], incorporating CHWs into the socio-ecological model–based mHealth intervention could substantially enhance its effectiveness. Future randomized behavioral clinical trials assessing whether the mHealth intervention may improve BP control independent of CHWs are warranted. In addition, the most popular educational modules completed by the study participants (eg, *Risk Factors*, *High BP*, and *PA*) were as anticipated, as these topics were closely aligned with and relevant to hypertension self-management. Furthermore, these results may reflect areas of CVH literacy that are of high interest to African American patients. Thus, digital health interventions should contextualize health education features to meet the learning priorities of African American patients as a form of digital health social justice [73].

Our study has several strengths. To our knowledge, this is the first study of its kind to use both integrated care health models and an mHealth app to improve hypertension management exclusively among African American patients. The few studies assessing the delivery of mHealth interventions by health care workers have largely been conducted in low- and middle-income countries [74]. Existing CHW-led interventions have revealed their effectiveness in managing chronic diseases, including hypertension, and in CVD risk reduction and demonstrated overall cost-effectiveness in minoritized racial and ethnic populations [75,76]. Our CHWs also served as digital health navigators to assist patients with the use of our mHealth app, consistent with the National Institute on Minority Health and Health Disparities research framework to advance patient digital agency and technology adoption [66,77]. In addition, CHWs in our study received evidence-based, intensive training on hypertension management and CVH promotion within the African American community, conducted SDOH-related social needs screening, and provided referrals to community resources. Our intervention is distinct in that the culturally tailored mHealth app uniquely served a dual purpose as an innovative educational tool for patients and CHWs to partner in improving BP control. Furthermore, our culturally tailored app with the adjunct support provided by CHWs allowed for an effective triad (patient-CHW-clinician) with reciprocal data transfer to enhance overall patient care and satisfaction. Another major strength was our integration of the user-centered and participatory design of the intervention with the inclusion of the prioritized population of African American patients with uncontrolled hypertension and their clinicians to better reflect their specific needs, sociocultural influences, and support systems [30,66].

### Limitations

This study is best understood in the context of its limitations. The main study limitations were the smaller sample size and attrition, which directly resulted from the impact of the COVID-19 pandemic and civil unrest that ensued after the death of Mr George Floyd. However, the clinically meaningful improvements in BP outcomes despite the negative impact of the COVID-19 pandemic on this study population and the disruptions in the administration of our study suggest that our intervention could potentially yield more robust reductions in BP in the absence of a public health emergency. Furthermore, our study design was single group and nonrandomized. Nonetheless, the intervention demonstrated feasibility, acceptability, and promising results in terms of improving hypertension self-management among African American patients receiving care at FQHCs. There was a lack of direct integration of the data collected from patients within the app with the EMR and clinicians. Finally, we did not assess comprehensive clinician-level data, including medication intensification and postintervention satisfaction. Future studies should explore more efficient SDOH screening with direct synchronization with the EMR and patient portals to allow for more streamlined communication among the patient-CHW-clinician triad [78].

### Conclusions

Our study suggests that a culturally tailored mHealth lifestyle intervention with adjunct support from a CHW could promote hypertension self-management with clinically meaningful improvement in BP among underresourced African American patients. Broader policy-level changes to incorporate mHealth tools and CHWs into care management teams may provide solutions to reduce the burden of CVD among African American patients. A future randomized efficacy trial of this intervention is warranted.

## Appendix

Multimedia Appendix 1 Patient recruitment materials, weekly visit activities form, moderator guide, summary of completed educational modules, patient app ratings, activity snapshots, types of needs identified, common services needed, and patient baseline characteristics.

## Abbreviations

BP: blood pressure
CBPR: community-based participatory research
CFIR: Consolidated Framework for Implementation Research
CHAT: Community Health Advocate Training
CHW: community health worker
CONSORT: Consolidated Standards of Reporting Trials
CVD: cardiovascular disease
CVH: cardiovascular health
DASH: Dietary Approaches to Stop Hypertension
EMR: electronic medical record
FAITH: Fostering African-American Improvement in Total Health!
FQHC: federally qualified health center
Health-ITUES: Health Information Technology Usability Evaluation Scale
H-SCALE: Hypertension Self-Care Activity Level Effects
mHealth: mobile health
NHLBI: National Heart, Lung, and Blood Institute
NIH: National Institutes of Health
PA: physical activity
PI: principal investigator
PRAPARE: Protocol for Responding to and Assessing Patients’ Assets, Risks, and Experiences
SDOH: social determinants of health

## Data Availability

The data supporting the study findings are available from the corresponding author (LPCB) upon reasonable request.
