# Supplementary material for: mHealth Intervention for Promoting Hypertension Self-management Among African American Patients Receiving Care at a Community Health Center: Formative Evaluation of the FAITH! Hypertension App
Source: JMIR Form Res. 2023 Jun 16;7:e45061. doi: 10.2196/45061 (PMC10337371; doi:10.2196/45061)
Supplement: Multimedia Appendix 1 [file formative_v7i1e45061_app1.pdf]

SUPPLEMENTAL MATERIAL

Mobile Health Intervention to Promote Hypertension Self-Management among African Americans Receiving Care at a Community Health Center: Formative Evaluation of the FAITH! Hypertension App

This supplement contains the following elements:

Supplemental Document 1. Phase 1 Patient Recruitment Material

Supplemental Document 2. Phase 2 Patient Recruitment Material

Supplemental Document 3. Weekly Visit Activities Form

Supplemental Document 4. Virtual Focus Group Moderator Guide

Supplemental Table 1. Summary of Completed Educational Modules

Supplemental Table 2. FAITH! Hypertension App Rating by Patients

Supplemental Figure 1. Overall NowPow© Activity Snapshot

Supplemental Figure 2. NowPow© Activity Snapshot by User

Supplemental Figure 3. Types of Needs Identified Among Pilot Participants

Supplemental Table 3. Ten Most Common Services Needed, Referrals Provided to FAITH! Patients, and Distance to Service Provider

Supplemental Table 4. Patient Baseline Characteristics by Study Completion Status

Supplemental Document 1. Phase 1 Patient Recruitment Material

African-Americans with High Blood Pressure Disease Needed for New Project

Mayo Clinic is seeking African-American men and women with High Blood Pressure to participate in a research study. The purpose of this study is to design and tailor a smartphone app to meet the needs of patients with High Blood Pressure.

You may be eligible to participate if:

- You are African-American
- You are a man or woman over 18 years old
- You have High Blood Pressure

This study will last for about 4-5 months and includes 2 focus groups (discussion meetings). We will provide you with $50 in cash cards and healthy meals at the focus groups. We will ask you to complete a questionnaire.

For more information about this study, please contact [redacted] at [redacted] or [redacted].

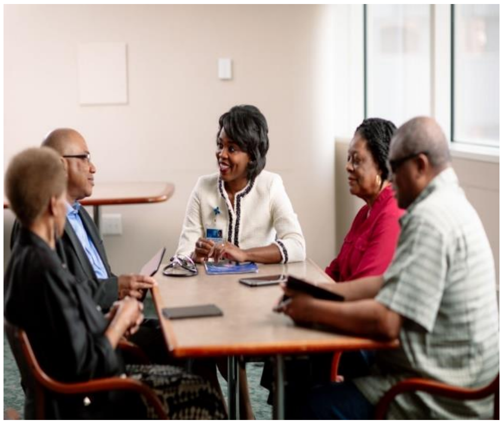

FAITH!

MAYO CLINIC

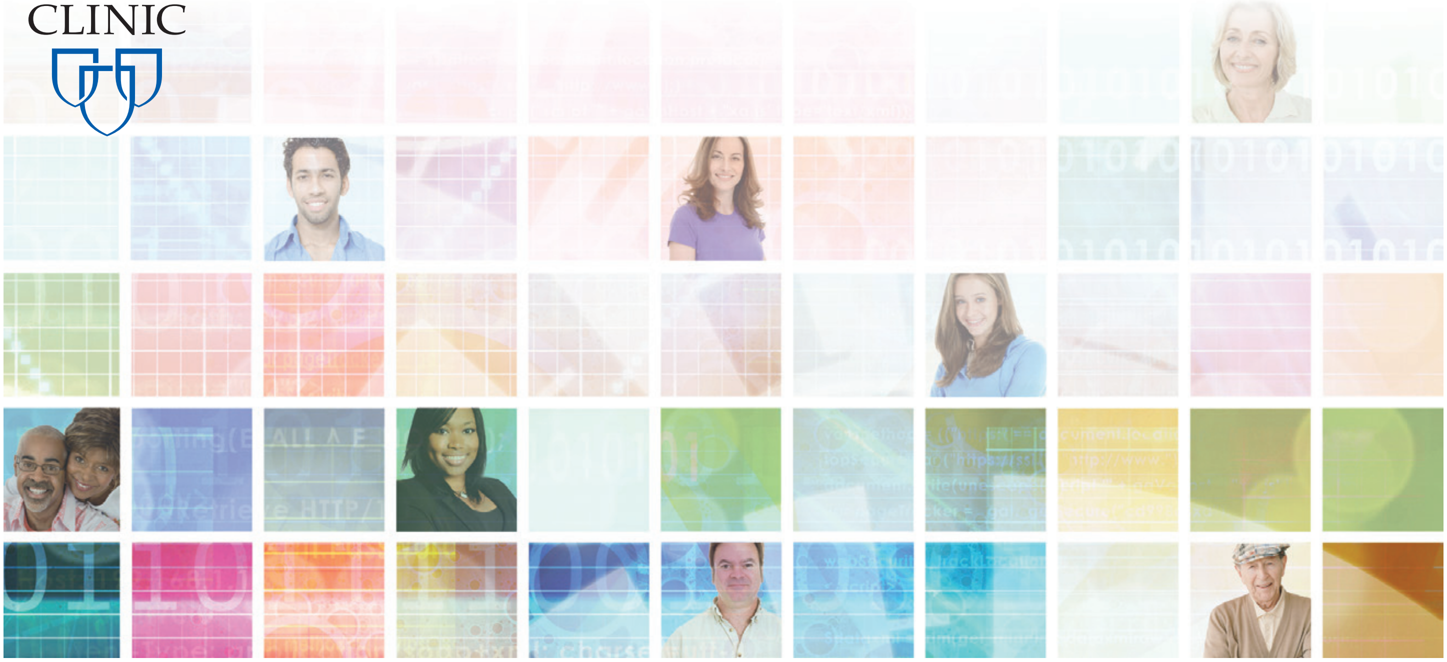

African-Americans with High Blood Pressure Needed for New Project

Mayo Clinic is seeking volunteers with high blood pressure, ages 18 and older, to participate in a research study. The purpose of this study is to use a smartphone app to help control your blood pressure.

You may be eligible to participate if:

- You are African American
- You are over 18 years old
- You have High Blood Pressure

Participation will last for 6 months, including 2 follow up sessions. A community health worker will also be involved in your care. You will be asked to complete a health assessment and survey at the start of the study and at follow up sessions. Participants will receive up to $100 for participating and a wireless blood pressure monitor.

For more information, contact a member of the study team at [redacted], and [redacted]

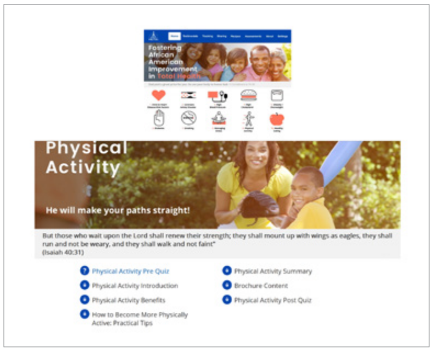

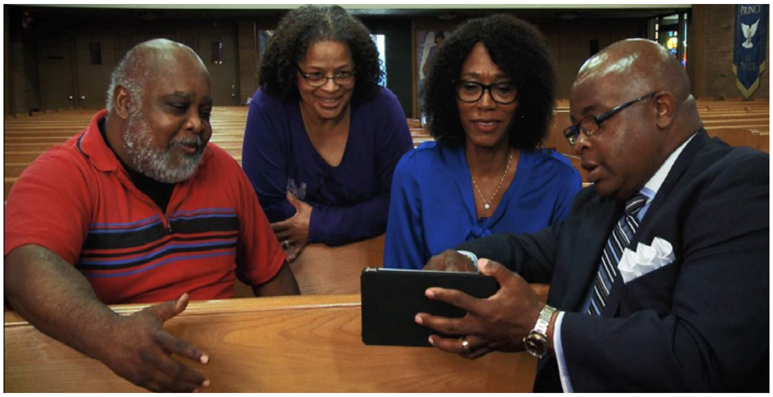

©2020 Mayo Foundation for Medical Education and Research

Supplemental Document 3. Weekly Visit Activities Form

FAITH! High Blood Pressure Project
CHW Activities Form

Date________________ Study ID: _______________

Patient Name:____

Address: ______________________________ City: ______________ Zip: ________

Home Phone: _____________________ Cell: _____________________ Work: _____________________

Primary Clinic:

- ☐ NorthPoint
- ☐ Open Cities

Primary Care Provider:____

Checklist

Medical Info: Average BP from patient home BP measurements

_____________/ ________________ mmHg

Please check known patient barriers patient would like addressed:

[ ] Transportation

[ ] Depression/Mental health referrals

[ ] Financial or other assistance related to insurance, food, housing, access to medication

[ ] Missed medical appointments

[ ] Assistance in understanding or following nutrition education already provided

[ ] Assistance in understanding medication adherence as prescribed

[ ] Other: ____

BP medical needs: (As directed by primary care provider)

BP medication review: (Review medications and assess adherence)

Social determinant needs identified:

Transportation	Housing
Depression/Mental Health	Medical bills
Insurance coverage	Utility repairs
Getting fresh, affordable food	Missed medical appointments
Paying for medications	Legal issues
Other: ______________________	Other: ______________________

Services provided:

Social Security Extra Help Program
Medical-Legal Partnership (MLP)
Health Care Assistance Program (HCAP)
Food stamps/WIC
Home Energy Assistance Program (HEAP)
Local food bank referral
Other: ______________________ Other: ______________________

Education reinforced: (Including review of modules completed, app use)
____

Progress: ____

____

Follow-up with primary care provider date: __________________

Next CHW visit date: __________________ Phone Meeting

Referrals pending: ______________________________

Comments:____
____
____
____

Signature Line: ______________________________ Date____________________

Supplemental Document 4. Virtual Focus Group Moderator Guide

NorthPoint Pilot Participant Focus Group Guide

INTRODUCTION & BACKGROUND

Hello! Thank you for participating in the FAITH! Hypertension Study and for taking the time to join us to discuss the FAITH! Hypertension App Program. My name is [redacted] and I'm from the MN Dept of Health. [redacted], also from MDH, will be taking notes. We are working with [redacted] from Mayo on using and evaluating the FAITH! app with NorthPoint patients.

We want to know what you like and don't like about using the FAITH! App. We will use the information and ideas gathered in these focus groups to make changes to the app or changes to how the app is used with clinic patients in the future.

My role is to ask questions, listen, and make sure everyone has a chance to share. To ensure a positive conversation we want to set a few ground rules:

- There are no right or wrong answers.
- Please feel free to share your point of view even if it differs from what others have said. Please listen respectfully as others share their ideas or views.
- We are interested in hearing from each of you. So I may call on you to respond. We just want to make sure all of you have a chance to share your ideas.

With your permission, I would like to record this session so we do not miss any important details. With that being said, it is important that when you speak, please speak up and only one person should talk at a time. The notes and audio will be used to develop a report, which will be used to guide changes made to the FAITH! App. Your privacy is very important to us. The report will not include any personal information from you and no one will be able to connect answers back to you, ensuring your confidentiality. Is everyone comfortable with me recording? (If not, recording will not be started).

First, let's find out some more about each other before starting. Let's go around and share your first name and your favorite summer activity? ______ let's start with you.

Thanks for sharing everyone! Now that we know a little more about one another, we can start our conversation.

FOCUS GROUPS QUESTIONS

We'd like to hear your thoughts on using the app to help manage your hypertension or high blood pressure:

1. How did you initially hear about the FAITH! app?
2. What was your experience like using the app? Was it easy or difficult? Why?
3. Overall, how satisfied have you been with the app?

Now we'd like to hear your thoughts on the information in the app:

4. Do you feel like the information in the app was useful for you?
5. Have you made any changes to your lifestyle while using the app? What kind of changes?
6. How would you describe your confidence in your ability to manage your hypertension or high blood pressure after using this app? (less confident, the same, more confident)
7. How would you describe your motivation to manage your hypertension or high blood pressure after using this app? (less motivated, the same, more motivated)
8. Do you have any suggestions for changes to the app or the FAITH! program?

Next, we'd like to hear from you about your experience working with your Community Health Worker:

9. What was your experience like with the Community Health Worker [redacted]?
10. What did you like most about working with a CHW in managing your blood pressure?
11. What did you like least about working with a CHW in managing your blood pressure?
12. In terms of your providers at NorthPoint, how would you have liked them involved with use of the app or the FAITH! program?
13. Is there anything else you would like to share about the app or your experience using the app?

Thank you for taking the time to talk with us this evening. We will be mailing you a gift card as a thanks for participating this evening.

Supplemental Table 1. Summary of Completed Educational Modules

	**Final Sample (N=7)**	**Enrolled Sample (N=16)**
**Variable**	**Number (%)**	
Average number of modules completed, mean (SD)	3.7 (4.6)	3.3 (4.5)
≥50% modules completed	3 (43)	6 (38)
**Education Modules**		
Risk Factors	3 (43)	6 (38)
High Blood Pressure	3 (43)	6 (38)
Physical Activity	3 (43)	6 (38)
Healthy Eating	3 (43)	6 (38)
Coronary Artery Disease	3 (43)	6 (38)
Cholesterol	3 (43)	6 (38)
Obesity	2 (29)	5 (31)
Diabetes	2 (29)	4 (25)
Smoking	2 (29)	4 (25)
Stress	2 (29)	4 (25)

Supplemental Table 2. FAITH! Hypertension App Rating by Patients

	**N (%)**
FAITH! App Helpful in Managing High Blood Pressure (0 to 10)	9.0 (1.9)
**FAITH! App Feature Rating**	
Home Page	
Excellent	4 (57)
Very Good	2 (29)
Good	1 (14)
Weekly Modules: Videos	
Excellent	4 (57)
Very Good	2 (29)
Good	1 (14)
Weekly Modules: Pre/Post Quizzes	
Excellent	4 (57)
Very Good	2 (29)
Good	1 (14)
Weekly Modules: Brochure Content	
Excellent	4 (57)
Very Good	2 (29)
Good	1 (14)
Blood Pressure Monitoring Log	
Excellent	4 (57)
Very Good	2 (29)
Good	1 (14)
Blood Pressures Dashboard	
Excellent	4 (57)
Very Good	2 (29)
Good	1 (14)
Tracking Log	
Excellent	4 (57)
Very Good	2 (29)
Good	1 (14)
Med List	
Excellent	4 (57)
Very Good	2 (29)
Good	1 (14)
Syncing of Omron BP Monitor to FAITH! App	
Excellent	4 (57)
Very Good	2 (29)
Good	1 (14)

Testimonials	
Excellent	4 (57)
Very Good	2 (29)
Good	1 (14)
Fitness Videos	
Excellent	4 (57)
Very Good	2 (29)
Fair	1 (14)
Sharing Board Posts by Mayo Clinic Team	
Excellent	4 (57)
Very Good	1 (14)
Good	2 (29)
Sharing Board	
Excellent	4 (57)
Very Good	1 (14)
Good	2 (29)
Recipes	
Excellent	4 (57)
Very Good	1 (14)
Good	1 (14)
Fair	1 (14)
FAITH! Speaker Bios	
Excellent	3 (43)
Very Good	2 (29)
Good	2 (29)
Settings	
Excellent	4 (57)
Very Good	1 (14)
Good	2 (29)
Bible Verses/Links to Bible Gateway Site	
Excellent	4 (57)
Very Good	1 (14)
Good	2 (29)
Smartphone Format/Accessibility	
Excellent	4 (57)
Very Good	1 (14)
Good	2 (29)
**FAITH! App Rating**	
Coverage of Information	
Excellent	5 (71)
Very Good	1 (14)

Good	1 (14)
Organization of Information	
Excellent	6 (86)
Good	1 (14)
Ease of Understanding Information	
Excellent	5 (71)
Very Good	2 (29)
Design and Appearance	
Excellent	4 (57)
Good	3 (43)
Ease of Navigation	
Excellent	4 (57)
Good	3 (43)
Ease of Use	
Excellent	4 (57)
Good	2 (29)
Fair	1 (14)
Integration of Features	
Excellent	5 (71)
Good	1 (14)
Fair	1 (14)
Cultural Appropriateness	
Excellent	4 (57)
Good	3 (43)
Recommend This Program to Other Patients/Family/Friends	
Yes	7 (100)

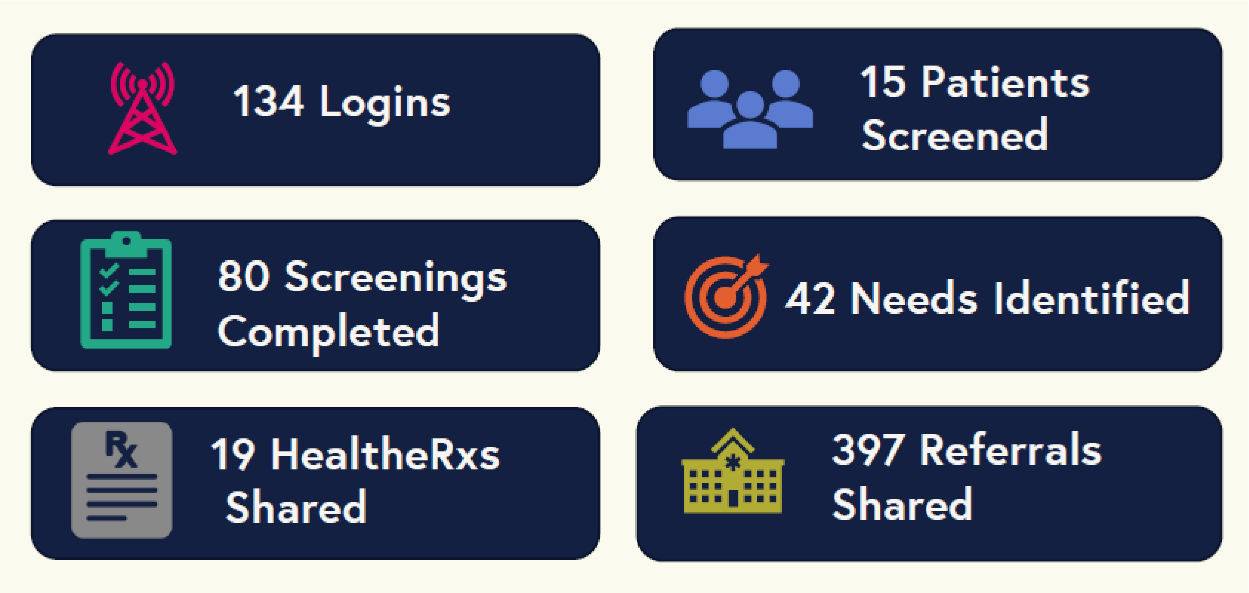

Supplemental Figure 1. Overall NowPow© Activity Snapshot

Activity Snapshot by User (5/10/2021 - 7/19/2021)

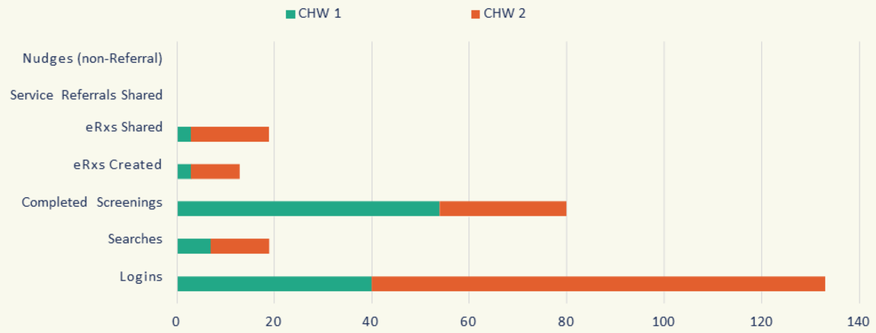

	Logins	Searches	Completed Screenings	eRxs Created	eRxs Shared	Service Referrals Shared	Nudges (non-Referral)
CHW 1	40	7	54	3	3	0	0
CHW 2	93	12	26	10	16	0	0

6 NOT FOR REDISTRIBUTION | @2021 NOWPOW. All rights reserved.

Supplemental Figure 2. NowPow© Activity Snapshot by User

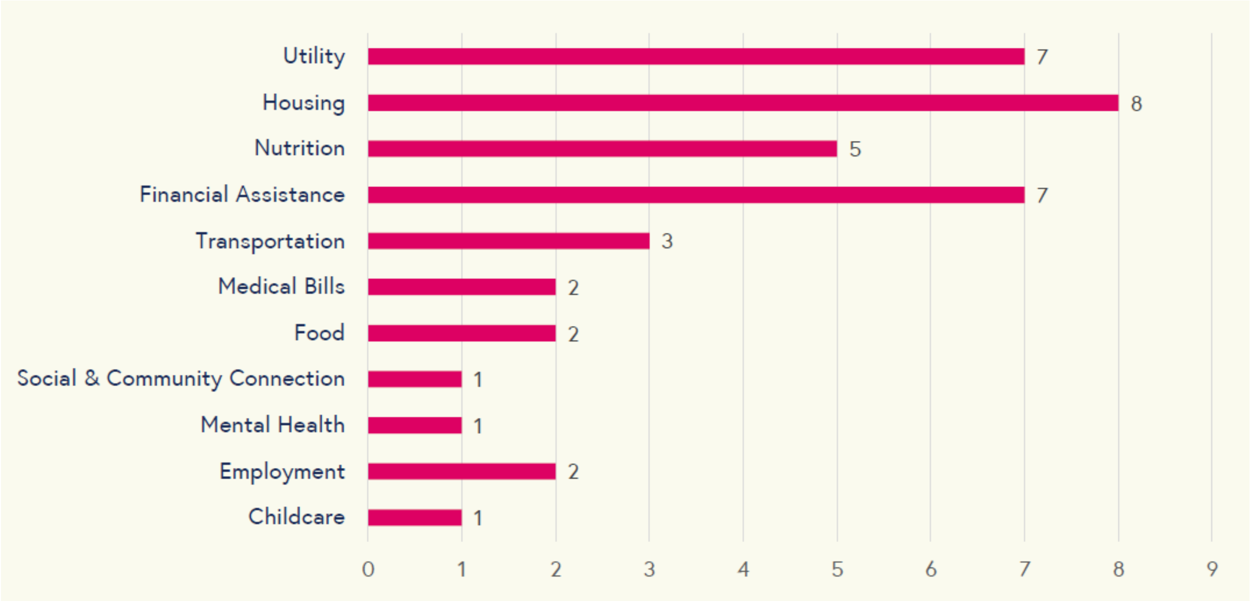

Supplemental Figure 3. Types of Needs Identified Among Pilot Participants

Supplemental Table 3. Ten Most Common Services Needed, Referrals Provided to FAITH! Patients, and Distance to Service Provider

10 Most Common Service Types	Referrals Shared	Average Distance to Service Provider (miles)
Rent and mortgage payment assistance	30	3.12
Income-based housing	29	3.95
Tenants' rights education	29	4.50
SNAP registration assistance	28	3.31
Utility payment assistance	28	3.55
Nutrition counseling	24	3.53
Temporary cash assistance for families	24	7.33
WIC registration assistance	24	4.82
Emergency-only financial assistance	21	4.77
Eviction and foreclosure prevention	20	6.30

Supplemental Table 4. Patient Baseline Characteristics by Study Completion Status

**Characteristics**	**Completed (N=7)**	**Not Completed (N=9)**	
	**Number (%)**		**P-value**
Age, years, mean (SD)	48.4 (12.3)	55.8 (12)	0.25
Gender			
Women	4 (57)	8 (89)	0.15
**Clinical Characteristics**			
Systolic BP, mmHg, mean (SD)[a]	149.7 (15.3)	143.7 (18.9)	0.51
Diastolic BP, mmHg, mean (SD)[a]	93.4 (8.9)	95.2 (15.3)	0.79
Body mass index, kg/m^2, mean (SD)	36.3 (9.1)	38.2 (14.3)	0.77
Hypertension diagnosis + Chronic condition	7 (100)	9 (100)	
**Social Determinants of Health**[b]			
Annual household income ≤ 200% of federal poverty level[d]	3 (60)	1 (100)	0.44
Has housing[d]	3 (50)	6 (75)	0.33
Worried about losing housing[d]	1 (33)	2 (40)	0.85
< High school degree[d]	0 (0)	1 (13)	0.37
≥ Part-time or full-time employment[d]	3 (50)	0 (0)	0.024
Medicaid and/or Medicare Insurance[d]	3 (50)	6 (86)	0.16
Unmet health-related social need over past year[d]			
Food[d]	4 (80)	1 (33)	0.19
Utilities[d]	4 (80)	1 (25)	0.1
Clothing[d]	2 (50)	2 (50)	1
Lack of Transportation	1 (14)	2 (22)	0.69
Social Integration < 5 times/week[d]	3 (50)	4 (57)	0.8
Stress[d]	5 (83)	6 (86)	0.91
Physical/emotional safety at home[d]	3 (75)	4 (50)	0.41
**Digital determinants of health**[c]			
Smartphone ownership	7 (100)	9 (100)	
Internet access beyond smartphone	6 (86)	8 (89)	0.85
Comfortable with mobile technology	4 (57)	7 (78)	0.38
Confidence using Internet to make health decisions	7 (100)	7 (78)	0.18

BP indicates blood pressure.

[a]Note blood pressure is last recorded within patient electronic medical record at time of eligibility screening.

[b]Adapted from the Protocol for Responding to & Assessing Patients' Assets, Risks & Experiences (PRAPARE) tool [63].

[c]Questions developed by study team based on domains outlined by Richardson et al [74].

[d]Some data are missing.
